# Cleaning of graphene surfaces by low-pressure air plasma

**DOI:** 10.1098/rsos.172395

**Published:** 2018-05-16

**Authors:** Phuong Viet Pham

**Affiliations:** SKKU Advanced Institute of Nano Technology (SAINT), Sungkyunkwan University (SKKU), Suwon, Gyeonggi-do 440-746, Republic of Korea

**Keywords:** graphene, CVD, cleaning, air-assisted plasma, roughness

## Abstract

The polymer residues still present on a chemical vapour-deposited graphene surface after its wet transfer by the poly(methyl methacrylate) method to the arbitrary substrates, tend to cause problems such as electrical degradation and unwanted intentional doping. In this study, by using an effective cleaning method for the graphene surface by air-assisted plasma, the graphene surface was cleaned significantly without damaging the graphene network, which resulted in the reduction (approx. 71.11%) of polymer residues on its surface. The analysis reveals that this approach reduced the D-band (impurities, polymer residues) formation while maintaining the π-bonding of the graphene, which affects conductivity. By characterizations of the optical microscope, Raman spectroscopy and atomic force microscopy, we obtained a significantly cleaner graphene surface (roughness of 4.1 nm) compared to pristine graphene (roughness of 1.2 nm) on a SiO_2_ substrate. In addition, X-ray photoelectron spectroscopy data revealed that the C1s peak of the air-assisted graphene film was higher than the one of a pristine graphene film, indicating that a cleaner graphene surface was obtained.

## 1. Introduction

Graphene, an sp^2^-plane-bonded carbon atom structure with honeycomb crystal lattice and a zero band gap semiconductor with mass-less charge carriers, has attracted huge research interest during the last few years owing to its anomalous properties such as very high carrier mobility, extremely high mechanical strength and optical transparency, electrical conductivity, chemical stability and thermal conductivity [1–8], and that is the reason graphene is being observed as a potential material for next-generation semiconductor devices that would replace silicon-based technology. Owing to being an atomically thin material, every atom of graphene has access to the surface that is directly responsible for its electronic and chemical activity. However, for many applications, graphene in pristine form cannot be used owing to the absence of band-gap and high sheet resistance [9]. The graphene synthesized by using thermal chemical vapour deposition (CVD) and other methods exhibits a lot of defects and polymer residues on the surface, which in turn, lowers its electrical conductivity.

Therefore, many previous studies have demonstrated how to remove poly(methyl methacrylate) (PMMA) residues from the graphene surface by the use of methods such as use of wet chemicals like acetone [10,11], cleaning by chloroform or toluene [12], by *N*-methyl-2-pyrrolidone [13], by diazonium salt [14]; a modified Radio Corporation of America (RCA) cleaning process and mechanically sweeping away the contamination [15]; oxygen plasma and reactive ion etching treatment for a short time [10,11,14]; mechanical method: an atomic force microscope (AFM) tip can remove all residue (theoretically without damaging the sample) in a contact mode [16]; annealing in high temperature [10,11,17,18]; current annealing [19] or by acetic acid [20]. However, these techniques involve either complicated wet chemistry or are limited to cleaning a local area only. A PMMA thin layer is known to modify graphene surface properties and to cause weak p-doping; however, removing the PMMA residue is more difficult than it seems. It cannot be dissolved with any known organic solvents. To obtain a clean surface, graphene samples are often thermally treated (150–400°C) to burn off the PMMA residue after a series of device processes.

Until now, there has been no effective method for complete removal of polymer residues, even using the chemical method (acid) or physical mechanism (Ar plasma cleaning) [21], or both of them. The existence of at least a little polymer residues or defects is inevitable because of the nature of the CVD graphene surface (such as defects, grain boundaries, no uniformity). For instance, the PMMA and contaminants could be attached at the edges of wrinkles, or defects in the size of graphene domain and grain boundaries [22], or bending of the graphene surface owing to the unpolished copper foil's high roughness surface and imperfect growth processing [23]. Consequently, the exploration of a new method of mitigation with removal of as much polymer residues as possible is necessary and desirable. Plasma treatment of graphene also proved helpful in cleaning of residue from the graphene surface and in fine-tuning the graphene properties [21,24]. For example, an Ar inductively coupled plasma (ICP) was used to clean the graphene surface before fabricating the high-performance field effect transistor device [21]; another report showed an efficient plasma method using hydrogen plasma reduction of ink-jet printed graphene oxide for fabrication of flexible graphene electrodes [24]. A strategy using air-assisted plasma is one such effective method for removing the polymer residues effectively, which is carried out in this study.

## 2. Experimental

### 2.1. Experimental set-up

A plasma ICP source with a copper coil assisted by an air-assisted plasma source is taken. The plasma cleaning has been demonstrated at the condition of 13.56 MHz for 30–300 s. First, SiO_2_ substrates were cleaned by wet cleaning (acetone (10 min), ethonol (10 min), IPA (10 min)), then cleaned by air-assisted plasma at a power of 18 W in 300 s and a flow rate at 49 ml min^−1^, while graphene/SiO_2_ was cleaned at 7 W in 30 s, and at 49 ml min^−1^. Before the experiment, the vacuum pressure of the air-assisted plasma chamber was adjusted to about 2.5 Pa. For the synthesis of the graphene layer: graphene films were synthesized on Cu foil with X-ray diffraction (XRD) characteristics, as shown in the electronic supplementary material, figures S1 and S2a, by the CVD method. A Cu foil with an area of 6 × 4 cm^2^ and thickness of 75 µm was placed into a CVD vacuum chamber with the quartz tube. First, the CVD chamber was cleaned by Ar (500 sccm) for 5 min and then filled with H_2_ gas at a flow rate of 10 sccm; the Cu foil was annealed for 2 h at a temperature of 1050°C in an H_2_ environment. Next, graphene was synthesized at 1050°C in the environment of H_2_/CH_4_ (10/20 sccm) for 30 min, after which the chamber was cooled down to room temperature with H_2_ gas (10 sccm) for 2 h.

### 2.2. Characterization

The following techniques were used for this purpose. Optical microscopy (OM) (AXIO, Carl Zeiss) was used to observe the morphology of the graphene surface and the SiO_2_ substrate before/after cleaning with air-assisted plasma. An AFM (Bruker Dimension Icon) (mode (tapping), cantilever tip (thickness: 4 µm, length: 125 µm, width: 40 µm, frequency: 320 kHz), spring constant (42 N m^−1^)) was used to measure the surface roughness of the graphene surface before/after cleaning with air-assisted plasma. X-ray photoelectron spectroscopy (XPS) (ESCALAB 250Xi, Thermo Scientific) with a Mg K*α* twin-anode source and with a take-off angle of 45° was used to characterize of C1s peaks of graphene films with the pressure in chamber calibration at 1–10 mbar and the pass energy of approximately 57 eV. Raman spectroscopy (514 nm, Ar^+^ ion laser, Renishaw, RM-1000 Invia) with an excitation energy of 2.41 eV was used for the characterization of the graphene films. An XRD (Ultima IV) was used to observe the orientations of the Cu foil.

## 3. Results and discussion

**Figure 1.**
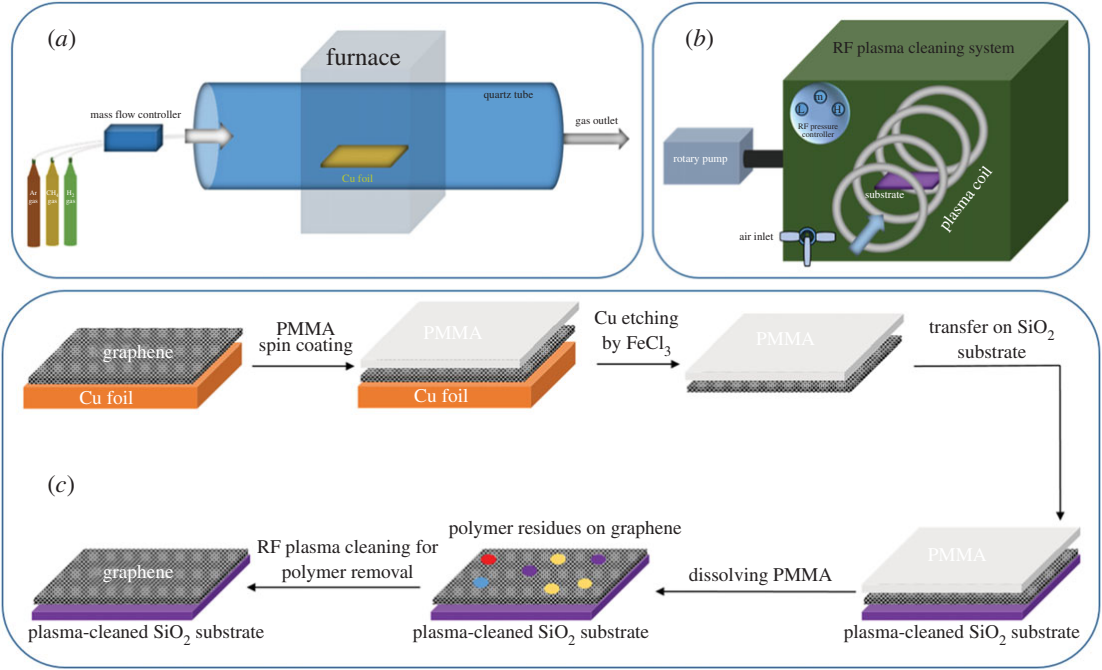
(*a*) Schematic of CVD system for graphene growth. (*b*) Schematic of the air-assisted plasma system for the cleaning process. (*c*) Sequences of graphene transfer by the PMMA method and air-assisted plasma cleaning.

As shown in figure 1*a*, a schematic of the CVD system for graphene growth is presented in detail in the experiment set-up. All samples were treated and cleaned with the air-assisted plasma system, as designed in figure 1*b*. Figure 1*c* shows a sequence of graphene transfer by the PMMA method and air-assisted plasma cleaning of the graphene surface located on the cleaned SiO_2_ substrate. In this study, low-pressure air plasma was used for the cleaning of graphene surfaces. Actually, the surface modification of flexible materials and electronic devices (e.g. graphene has good mechanical and flexible properties) has been well investigated in the previous reports [25,26].

**Figure 2.**
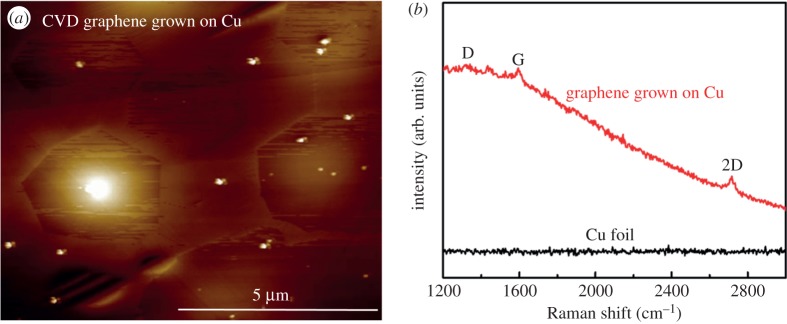
(*a*) AFM image of CVD graphene grown on a Cu foil. (*b*) Raman spectra of the poly crystal Cu foil and graphene/Cu.

The OM images of CVD graphene grown on a Cu foil with graphene domains showed white points (in the electronic supplementary material, figure S2b,c) which indicated impurities on it during CVD graphene growth. Besides a hexagonal shape, the graphene domains also were pentagonal with non-symmetric morphology, as shown in the AFM image in figure 2*a*. For further investigation, the Raman spectra were carried out for the as-received Cu foil and the graphene grown on Cu (figure 2*b*). In the case of the as-received Cu foil, Raman data showed no signal, while the D, G and 2D peaks presented in the case of graphene/Cu as typical fingerprints of graphene identification.

**Figure 3.**
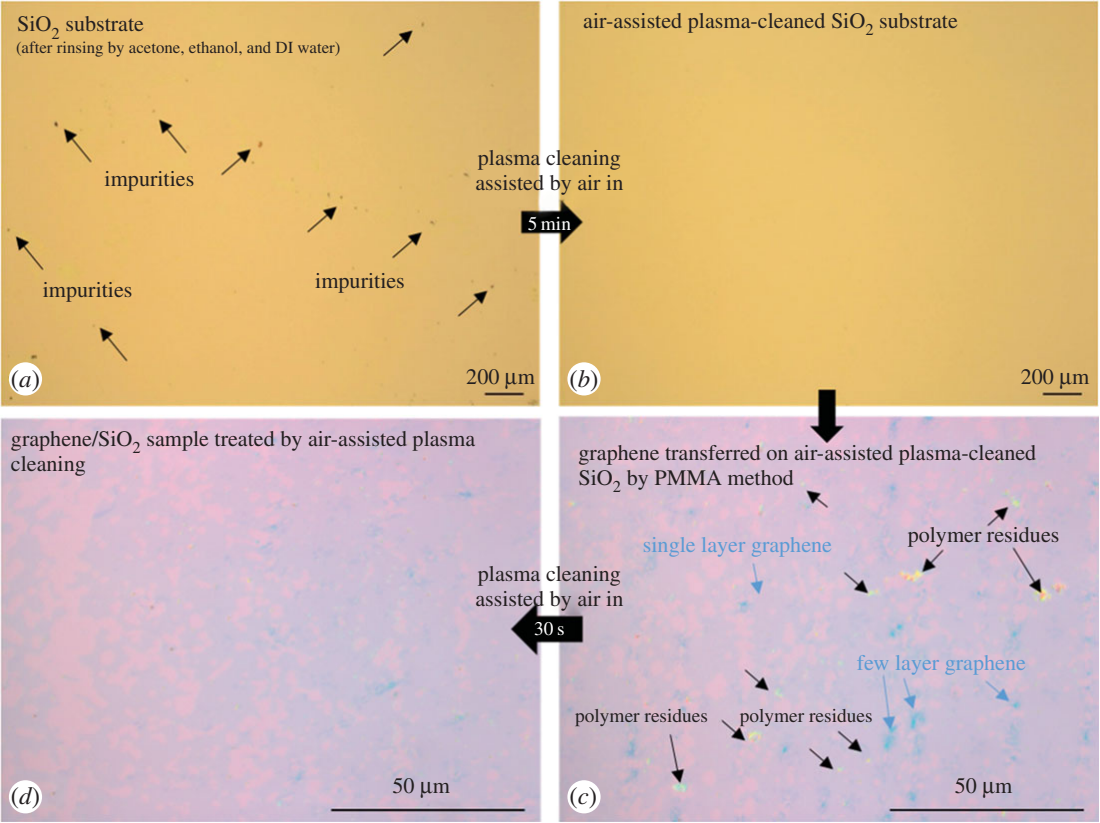
OM images of a pristine SiO_2_ substrate before (*a*) and after (*b*) air-assisted plasma cleaning; graphene transferred on to air-assisted plasma-cleaned SiO_2_ substrate (*c*) and graphene/SiO_2_ sample treated by air-assisted plasma cleaning (*d*).

The SiO_2_ wafer even packaged in the box from the company, however, still shows a lot of tiny impurities on its surface. Consequently, further cleaning is necessary by wet cleaning (acetone, ethanol and deionized (DI) water) and dry cleaning (air-assisted plasma) to obtain an ultra-clean SiO_2_ surface. Before transferring the graphene/Cu on to the SiO_2_ substrate, the SiO_2_ substrates were perfectly cleaned by air-assisted plasma for 5 min for removing impurities on its surface, which still existed after rinsing by acetone, ethanol and DI water for 20 min with each chemical, as shown in the OM images in figure 3*a,b*. After obtaining the ultra-clean SiO_2_ surface, graphene/Cu was transferred onto it by a kind of popular polymer named PMMA (figure 3*c*). Actually, the PMMA chemical still remained as polymer residues on graphene/SiO_2_ even after acetone rinsing (20 min) owing to weak van der Waal interactions and chemical bonding at the interface between the PMMA and graphene films. Therefore, it requires a cleaning process to remove as much polymer residues as possible. Consequently, a very clean graphene surface on SiO_2_ was obtained after 30 s of air-assisted plasma treatment (figure 3*d*).

**Figure 4.**
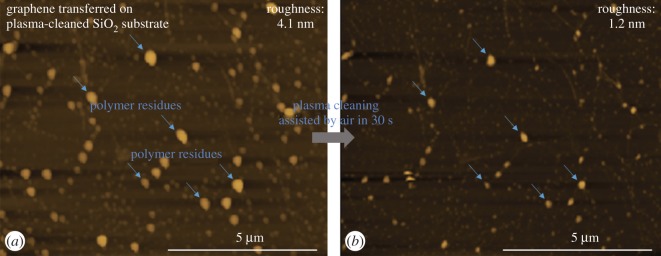
AFM images at the same position of the graphene/SiO_2_ sample before (*a*) and after (*b*) air-assisted plasma cleaning.

To investigate this cleaning effect, the AFM data were taken for investigation on the graphene/SiO_2_ surface before and after air-assisted plasma cleaning at the same position (figure 4). As a result, after plasma treatment, the roughness of the graphene surface decreased from 4.1 nm to 1.2 nm, about a 71.11% reduction.

**Figure 5.**
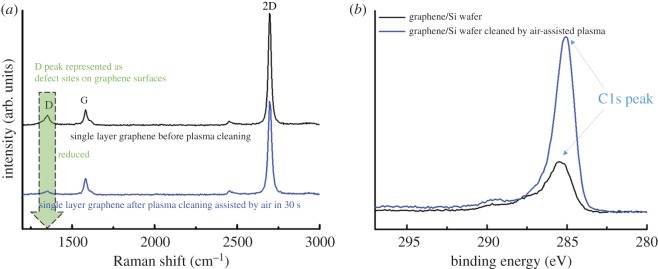
(*a*) Raman spectra of the graphene film before and after air-assisted plasma cleaning, (*b*) XPS spectra of the C1s peak of the graphene/Si wafer before and after air-assisted plasma cleaning.

A demonstration by Raman spectra was carried out for the cleaning effect. The Raman data (as shown in figure 4) were taken and obtained from the average value of six various spots on the graphene sample before and after air-assisted plasma treatment. The D peak presents as impurities and polymer residues reduced after air-assisted plasma cleaning of the graphene surface, without disordering of the graphene network (figure 5*a*). The XPS spectra were also studied in two cases of pristine graphene/Si and air-plasma cleaned-graphene/Si (figure 5*b*). The C1s peak of air-plasma-cleaned graphene/Si showed a very high peak compared with pristine graphene/Si, indicating the attainment of a cleaner graphene surface.

## 4. Conclusion

By an effective cleaning method for a graphene surface by air-assisted plasma, the graphene surface was cleaned significantly without disordering the graphene network, which results in reduction (approx. 71.11%) of polymer residues on its surface. Analysis reveals that this approach reduced the D-band (impurities, polymer residues) formation, which affects conductivity. By characterizations of OM and AFM images, and Raman spectroscopy, we obtained a significantly cleaner graphene surface (roughness of 1.2 nm) compared to pristine graphene (roughness of 4.1 nm) on the SiO_2_ substrate. In addition, XPS data revealed that the C1s peak of the air-assisted graphene film was higher than the one of a pristine graphene film, indicating that a cleaner graphene surface was obtained. We believe that this cleaning method could be very useful for fabrication in graphene-based electronic applications in the future.

## Supplementary Material

Viet Phuong Pham_figures_ESM

## Data accessibility

All the data are provided in the main text and the electronic supplementary material.

## References

[1] Novoselov KS, Geim AK, Morozov SV, Jiang D, Zhang Y, Dubonos SV, Grigorieva IV, Firsov AA 2004 Electric field effect in atomically thin carbon films. Science 306, 666–669. (doi:10.1126/science.1102896) 15499015 10.1126/science.1102896

[2] Novoselov KS, Geim AK, Morozov SV, Jiang D, Katsnelson MI, Grigorieva IV, Dubonos SV, Firsov AA 2005 Two-dimensional gas of massless Dirac fermions in graphene. Nature 438, 197–200. (doi:10.1038/nature04233) 16281030 10.1038/nature04233

[3] Dean CR et al. 2010 Boron nitride substrates for high-quality graphene electronics. Nature Nanotech. 5, 722–726. (doi:10.1038/nnano.2010.172) 10.1038/nnano.2010.172 20729834

[4] Pham VP, Jang HS, Whang D, Choi JY 2017 Direct growth of graphene on rigid and flexible substrates: progress, applications and challenges. Chem Soc. Rev. 46, 6276–6300. (doi:10.1039/c7cs00224f) 28857098 10.1039/c7cs00224f

[5] Pham VP et al. 2017 Chlorine-trapped CVD bilayer graphene for resistive pressure sensor with high detection limit and high sensitivity. 2D Materials 4, 025049 (doi:10.1088/2053-1583/aa6390)

[6] Pham VP, Mishra A, Yeom GY 2017 The enhancement of Hall mobility and conductivity of CVD graphene through radical doping and vacuum annealing. RSC Adv. 7, 16 104–16 108. (doi:10.1039/c7ra01330b)

[7] Pham VP, Kim KH, Jeon MH, Lee SH, Kim KN, Yeom GY 2015 Low damage pre-doping on CVD graphene/Cu using a chlorine inductively coupled plasma. Carbon 95, 664–671. (doi:10.1016/j.carbon.2015.08.070)

[8] Kim KN, Pham VP, Yeom GY 2015 Chlorine radical doping of a few layer graphene with low damage. ECS J. Solid State Sci. Technol. 4, N5095–N5097. (doi:10.1149/2.0141506jss)

[9] Brenner DW, Shenderova OA, Harrison JA, Sttuart JS, Ni B, Sinnott SB 2002 A second-generation reactive empirical bond order (REBO) potential energy expression for hydrocarbons. J. Phys. Condens. Matter 14, 783 (doi:10.1088/0953-8984/14/4/312)

[10] Mcevoy N, Nolan H, Kumar NA, Hallam T, Duesberg GS 2013 Functionalization of graphene surfaces with downstream plasma treatments. Carbon 54, 283–290. (doi:10.1016/j.carbon.2012.11.040)

[11] Peltekis N, Kumar S, Mcevoy N, Lee K, Weidlich A, Duesberg GS 2012 The effect of downstream plasma treatments on graphene surfaces. Carbon 50, 395–403. (doi:10.1016/j.carbon.2011.08.052)

[12] Niranan P, Marco S, Alberto D, Athanasia A 2011 Effect of solvents on the dynamic behavior of poly(methyl methacrylate) film prepared by solvent casting. J. Mater. Sci. 46, 5044–5049. (doi:10.1007/s10853-011-5424-9)

[13] Xuelei L et al. 2011 Toward clean and crackles transfer of graphene. ACS Nano 5, 9144–9153. (doi:10.1021/nn203377t) 21999646 10.1021/nn203377t

[14] Xiao YF, Ryo N, Li CY, Katsumi T 2010 Effects of electron-transfer chemical modification on the electrical characteristics of graphene. Nanotechnology 21, 475208 (doi:10.1088/0957-4484/21/47/475208) 21030765 10.1088/0957-4484/21/47/475208

[15] Chou SY, Krauss PR, Renstrom PJ 1996 Nanoimprint lithography. J. Vac. Sci. Technol. B 14, 4129–4133. (doi:10.1116/1.588605)

[16] Goosens AM, Calado VE, Barreiro A, Watanabe K, Taniguchi T, Vandersypen LMK 2012 Mechanical cleaning of graphene. Appl. Phys. Lett. 100, 073110 (doi:10.1063/1.3685504)

[17] Yung CL, Chun CL, Chao HY, Chuanhong J, Kazu S, Po WC 2012 Graphene annealing: how clean can it be? Nano Lett. 12, 414–419. (doi:10.1021/nl203733r) 22149394 10.1021/nl203733r

[18] Wei SL, Chang TN, John TLT 2014 What does annealing do to metal-graphene contacts? Nano Lett. 14, 3840–3847. (doi:10.1021/nl500999r) 24912079 10.1021/nl500999r

[19] Moser J, Barreiro A, Bachtold A 2007 Current-induced cleaning of graphene. Appl. Phys. Lett. 91, 163513 (doi:10.1063/1.2789673)

[20] Her M, Beams R, Novotny L 2013 Graphene transfer with reduced residue. Phys Lett. A 377, 1455–1458. (doi:10.1016/j.physleta.2013.04.015)

[21] Lim YD, Lee DY, Shen TZ, Ra CH, Choi JY, Yoo WJ 2012 Si-compatible cleaning process for graphene using low-density inductively coupled plasma. ACS Nano 6, 4410–4417. (doi:10.1021/nn301093h) 22515680 10.1021/nn301093h

[22] Duong DL et al. 2012 Probing graphene grain boundaries with optical microscopy. Nature 490, 235–239. (doi:10.1038/nature11562) 23034653 10.1038/nature11562

[23] Han GH, Gunes F, Bae JJ, Kim ES, Chae SJ, Shin HJ, Choi JY, Pribat D, Lee YH 2011 Influence of copper morphology in forming nucleation seeds for graphene growth. Nano Lett. 11, 4144–4148. (doi:10.1021/nl201980p) 21863812 10.1021/nl201980p

[24] Homola T, Pospisil J, Krumpolec R, Soucek P, Dzik P, Weiter M, Cernak M 2018 Atmospheric dry hydrogen plasma reduction of inkjet-printed flexible graphene oxide electrodes. ChemSusChem 11, 1–8. (doi:10.1002/cssc.201702139) 10.1002/cssc.201702139 29356373

