# Supplementary material for: Cleaning of graphene surfaces by low-pressure air plasma
Source: R Soc Open Sci. 2018 May 16;5(5):172395. doi: 10.1098/rsos.172395 (PMC5990796; doi:10.1098/rsos.172395)
Supplement: Viet Phuong Pham_figures_ESM [file rsos172395supp1.pdf]

Supporting Information

Cleaning of Graphene Surface Assisted by Air Plasma

Viet Phuong Pham

SKKU Advanced Institute of Nano Technology (SAINT), SKKU, Suwon, Gyeonggi-do 440-746, Republic of Korea

Email: pvphuong85@ibs.re.kr

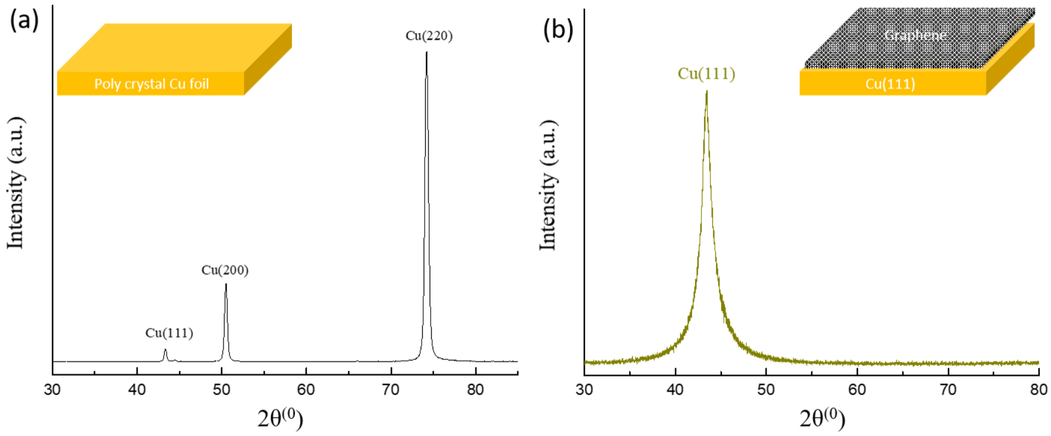

Figure S1. XRD data of poly-crystal Cu foil as-received (a) and graphene/Cu(111) foil after CVD growth.

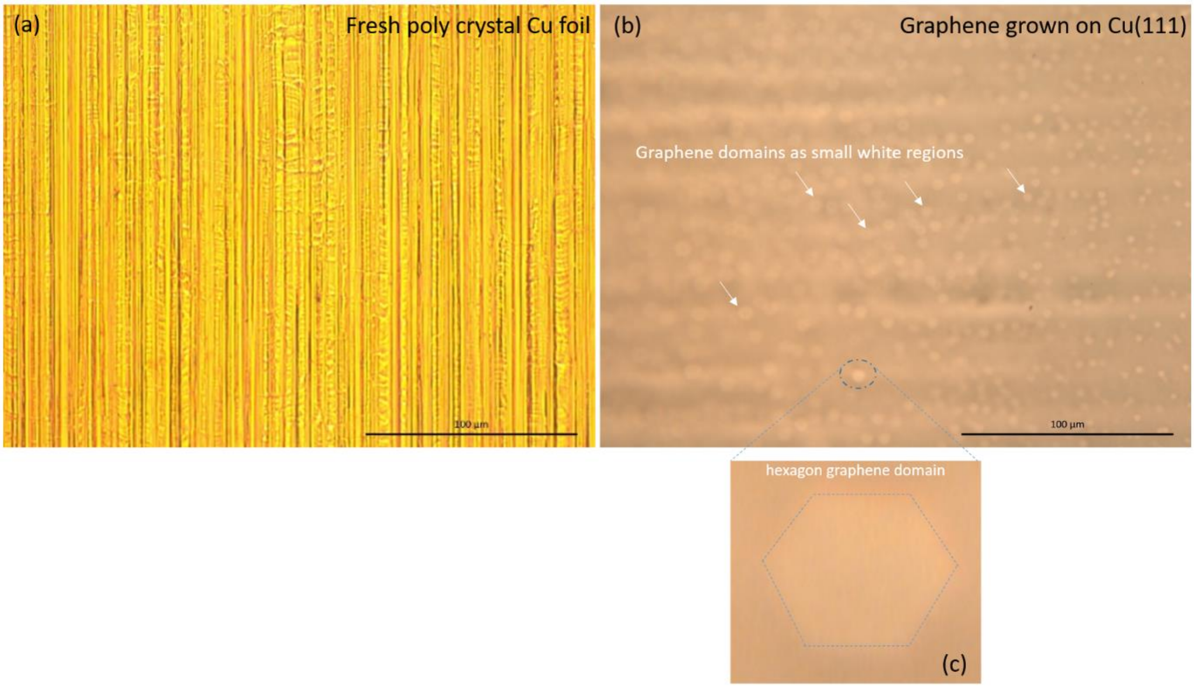

Figure S2. OM images of fresh poly-crystal Cu foil (a), graphene/Cu(111) foil grown by CVD method (b), and a typical hexagonal graphene domain (c) from figure (b).
